# Drug repositioning for non-small cell lung cancer by using machine learning algorithms and topological graph theory

**DOI:** 10.1186/s12859-015-0845-0

**Published:** 2016-01-11

**Authors:** Chien-Hung Huang, Peter Mu-Hsin Chang, Chia-Wei Hsu, Chi-Ying F. Huang, Ka-Lok Ng

**Affiliations:** Department of Computer Science and Information Engineering, National Formosa University, Hu-Wei, 63205 Taiwan; Division of Hematology and Oncology, Department of Medicine, Taipei Veterans General Hospital; Faculty of Medicine, National Yang Ming University, Taipei, 112 Taiwan; Institute of Biopharmaceutical Sciences, National Yang-Ming University, Taipei, 112 Taiwan; Department of Bioinformatics and Medical Engineering, Asia University, Taichung, 41354 Taiwan; Department of Medical Research, China Medical University Hospital, China Medical University, Taichung, 40402 Taiwan

**Keywords:** Non-small cell lung cancer, Drug repositioning, Microarray data analysis, Machine learning algorithm, Topological parameters, Protein-protein interactions, Enrichment analysis, Connectivity Map

## Abstract

**Background:**

Non-small cell lung cancer (NSCLC) is one of the leading causes of death globally, and research into NSCLC has been accumulating steadily over several years. Drug repositioning is the current trend in the pharmaceutical industry for identifying potential new uses for existing drugs and accelerating the development process of drugs, as well as reducing side effects.

**Results:**

This work integrates two approaches - machine learning algorithms and topological parameter-based classification - to develop a novel pipeline of drug repositioning to analyze four lung cancer microarray datasets, enriched biological processes, potential therapeutic drugs and targeted genes for NSCLC treatments. A total of 7 (8) and 11 (12) promising drugs (targeted genes) were discovered for treating early- and late-stage NSCLC, respectively. The effectiveness of these drugs is supported by the literature, experimentally determined in-vitro IC_50_ and clinical trials. This work provides better drug prediction accuracy than competitive research according to IC_50_ measurements.

**Conclusions:**

With the novel pipeline of drug repositioning, the discovery of enriched pathways and potential drugs related to NSCLC can provide insight into the key regulators of tumorigenesis and the treatment of NSCLC. Based on the verified effectiveness of the targeted drugs predicted by this pipeline, we suggest that our drug-finding pipeline is effective for repositioning drugs.

## Background

Lung cancer is the leading cause of death globally [1] and non-small cell lung cancer (NSCLC) accounts for more than 85 % of all lung cancer cases; adenocarcinoma is the most common subtype. Many efforts have been made to development treatments for NSCLC, and they depend on finding suitable drugs for treating NSCLC within an effective time and at reasonable cost.

Drug repositioning by the Food and Drug Administration (FDA) involves approving drugs with known side effects; it has become a major trend and seen some success. Stachnik et al. [2] showed that bisphosphonates can potentially be repurposed for the prevention and adjunctive therapy of HER1-driven cancers (such as NSCLC and breast cancers). Having constructed a drug-disease bipartite network, Chen et al. [3] utilized two inference methods, ProbS and HeatS, to predict direct drug-disease associations based on node degree in the network. Lee et al. [4] integrated the shared neighborhood scoring algorithm with a database of disease indications, drug development, and associated proteins, to identify new indications for known FDA-approved drugs. In earlier studies [5, 6], based on PPI (protein-protein interaction) community, we established a systematic strategy for identifying potential drugs and target genes for treating NSCLC, which can be extended in several respects that are addressed in the present study. Those two previous studies did not use the four features of machine learning algorithms that are used herein, and were proposed in our work in 2015 on the prediction of cancer proteins [7].

The machine learning method and the topological properties of biological networks have been used separately to identify cancer-related genes. For example, Bull et al. [8] utilized proteins’ hydrophobicities, in vivo half-lives, propensity for being membrane-bound and the fraction of non-polar amino acids as features in the Random Forest classifier to predict drug targets. Carson et al. [9] utilized topological metrics, such as betweenness centralities, neighborhood connectivity and radiality, as features and used an alternating decision tree (ADTree) classifier to identify disease-associated genes. Many works on identifying repositioned drugs have been based on various computational methods, such as mapping gene expression profiles using drug response profiles [10–14], the use of side-effect-based similarities [15–17], heterogeneous network clustering [18], and the graph-based inference method [19–22]. However, most of these methods are either disease-centric or drug-centric. To the best of the authors’ knowledge, few works have addressed the problem of drug repositioning by integrating machine learning methods, graph theory and meta-analysis. This work integrates two state-of-art methods - machine learning [7] and the graphing of topological properties [23] - to develop a new pipeline to identify potential therapeutic drugs and targeted genes for treating NSCLC.

In solving the targeted drug problem, the following issues must be addressed. First, different individuals may correspond to different sets of differentially expressed genes. Second, cancer is a heterogeneous disease: different stages of cancer require different drug targets and involve stage-specific cancer-associated genes. Third, the results of microarray profiling vary from study to study and a rigorous method is required to solve this problem. Fourth, the reliability of drug finding remains to be verified.

This study deals with the above four issues. First, to reduce the effect of biological heterogeneity among different individuals, tumor/adjacent non-tumor pairwise arrays for NSCLC were used, allowing pairwise statistical testing. Second, the samples were grouped into early-stage and late-stage samples. Third, meta-analysis was carried out to integrate multiple microarray profiles and results. Finally, potential drugs were validated by performing biochemical assays and with reference to the literature.

## Methods

**Fig. 1:**
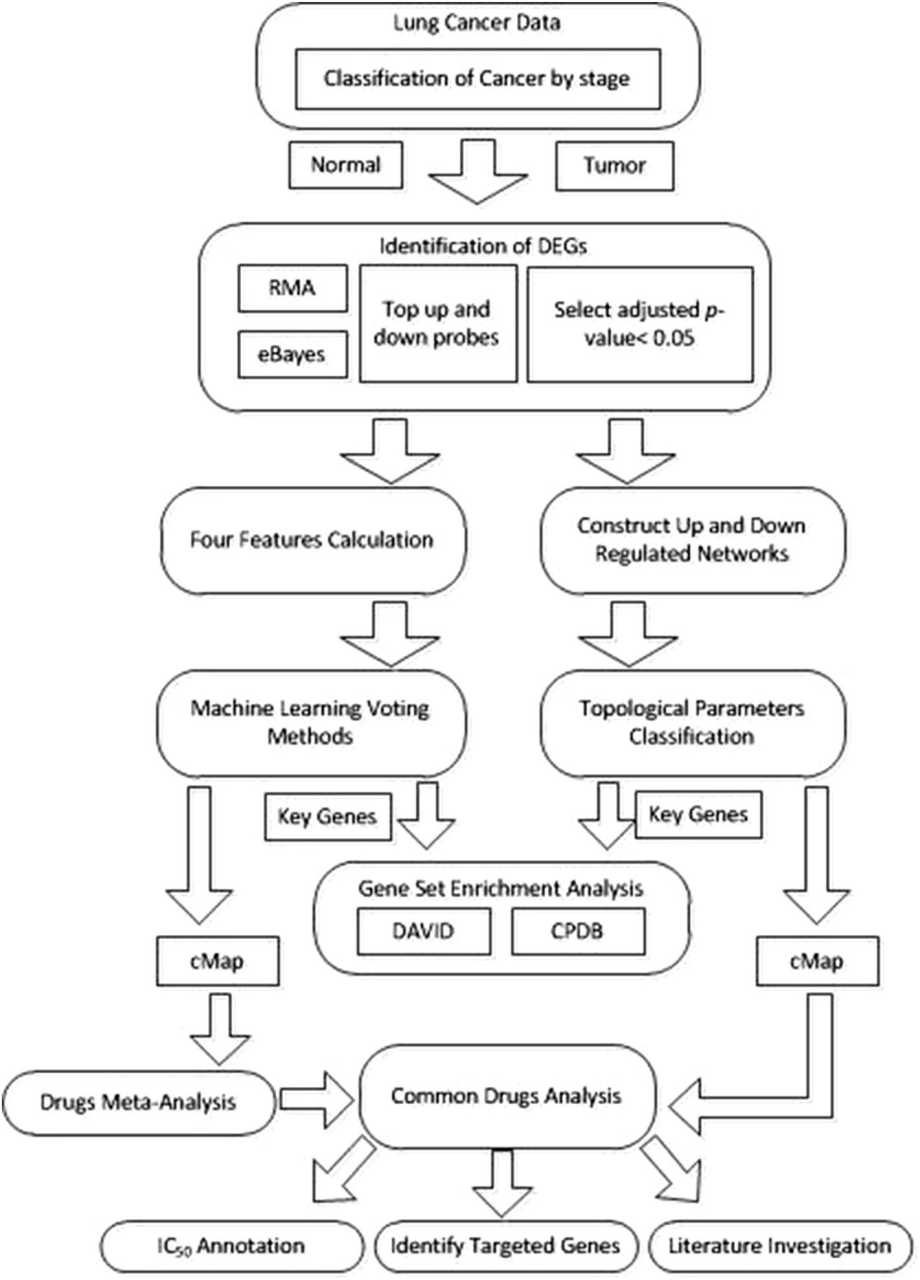
Workflow of this study, which consists of (1) identification of DEGs, (2) machine learning approach, (3) topological parameter-based classification, (4) common pathway analysis, (5) common drug analysis and (6) effectiveness verification

Cancer is a multistage process that arises from mutations of genetic sequences; early- and late- stage cancer-associated genes potentially differ considerably. This work elucidates a strategy for identifying stage-specific potential drugs for treating NSCLC based on an integrated analysis based on microarray profiling. This work proposes an *in silico* strategy for narrowing down the search for lung cancer genes. Figure 1 presents the workflow.

Microarray data for lung cancer were firstly separated into the early- and late-stage data. Two-pair tests (based on normal and cancer tissues from the same patient) were performed to identify differentially expressed genes (DEGs). A Robust Multi-array Average (RMA) was utilized to normalize gene expression, and eBayes analysis was then performed on the results thereof. DEGs were predicted using an adjusted *p*-value of 0.05. The selected DEGs were divided into two groups - an up-regulated group and a down-regulated group - based on the fold-changes (FC) in gene expression. These selected DEGs are separately filtered using machine learning classifiers and graph theory, and two corresponding sets of key genes are then derived. Gene set enrichment analysis and pathway analysis were then conducted on the two sets of key genes, and drug-gene interaction databases and the Connectivity Map (cMap) were used to identify potential drugs (with cMap *p*-value <0.1 and enrichment score <0) for treating NSCLC. The common enriched pathways and drugs that were returned by both machine learning algorithms and the classification of topological parameters were further investigated. The predictions of targeted drugs were confirmed by IC_50_ experiments, a review of the literature and clinical trials. Finally, the targeted genes were prioritized for reference.

### Input datasets

The input data herein were taken from previous work [6] to enable this study to be compared with [6]. The microarray data for lung cancer were downloaded from GEO [24] and summarized in Table 1. The microarray datasets consist of data from experiments GSE7670 [25] and GSE10072 [26], which were conducted using the HG-U133A array; and data from experiments GSE19804 [27] and GSE27262 [28], which were performed using HG-U133 plus 2.0 chip.

**Table 1:** Summary of microarray datasets

GEO ID	Organization name	Number of samples (Early-stage)	Number of samples (Late-stage)
GSE7670	Taipei Veterans General Hospital	8	11
GSE10072	National Cancer Institute, NIH	15	9
GSE19804	National Taiwan University	35	13
GSE27262	National Yang Ming University	25	n/a

To reduce the effect on integrating of biological heterogeneity among individuals, normal and cancer tissues were taken from each patient. Two-pair tests (on these normal and cancer tissues are taken from the same patient) were performed to identify differentially expressed genes (DEGs). Samples were divided into early- and late-stage samples. Early-stage samples were taken from patients with stage I, IA or IB cancer, while late-stage data were obtained from patients with stages III or IV cancer [6].

### Microarray data analysis

In this study, the publicly available microarray data analysis package *Bioconductor* was used to identify DEGs among a large number of gene expressions. Based on whether the log base 2 of the fold-change (FC) values for gene expression, log_2_FC, was less than or greater than zero, the selected DEGs were divided into two groups - up-regulated (up probes in Fig. 1) and down-regulated (down probes in Fig. 1), respectively. The FC value of any gene expression level with a fold change value of less than 5.64 was set to 5.64 to facilitate the cMap [29] search.

### Machine learning algorithms

In the previous study [7], we developed a simple and effective machine learning method, based on domain-domain interactions (DDI), weighted domain frequency score (DFS) and cancer linker degree data (CLD) to predict cancer proteins. We used the one-to-one interaction model to quantify the likelihood that was a cancer-specific DDI; the weighted DFS feature is used to measure the propensity of a domain to be present in cancer and non-cancer proteins, and the CLD feature is defined to identify the partners with which cancer and non-cancer proteins interact. The machine learning algorithms was implemented in the Weka software tool, and a ten-fold cross-validation test was used to train the supervised model. Based on our previous studies [30, 31], a balanced data set typically provides better performance than an unbalanced one, so, the machine learning algorithms were trained using positive and negative datasets that contained equal numbers of data.

Experimental results revealed that the proposed machine learning method identified cancer proteins with relatively high hit ratios (about 80 %). Five classifiers – three with the highest F1 values – the LMT, SimpleCart and J48 algorithms, and two with the highest AUC values – the LWL and Ridor algorithms, were used to identify potential cancer genes under strictly uniform voting, meaning that only a protein that was predicted by all five classifiers to be a cancer protein was considered. In the machine learning approach, the up- (down-) regulated DEGs in each microarray data are processed individually for each microarray.

### Classification of topological parameters

The topological features provide valuable information for identifying crucial genes and clusters in a biological network. Recently, we proposed the identification of critical nodes for a network using topological parameters [23]. The five classified groups are: group 1: degree centrality; group 2: betweenness centrality; group 3: bridging centrality; group 4: closeness centrality and eccentricity centrality; group 5: clustering coefficient, brokering coefficient and local average connectivity. This classification enables nodes to be ranked by their topological importance in the networks. To apply topological parameter classification in this study, common up- (down-) regulated DEGs for the microarray datasets must be firstly extracted. Next, for early- and late-stage NSCLC, the corresponding up- (down-) regulated network was constructed by using the common DEGs for all microarray datasets and their neighbors in protein-protein interactions. The up- and down-regulated networks for early- and late-stage NSCLC are inputs for the topological parameter-based classification.

### Enrichment analysis of gene set

Given a gene list, DAVID [32] performs batch annotation and GO [33] term enrichment analysis to highlight the most relevant GO terms. In contrast, the ConsensusPathDB (CPDB) [34] resource performs gene set analysis and metabolite set analysis. To find the enriched pathways of the proposed genetic signature for NSCLC, an over-representation pathway analysis was performed using both DAVID and CPDB using a *p*-value threshold of 0.05. Significant pathways were ranked by *p*-value. Both tools were utilized in this stage for cross-verification.

### Potential target genes and drug discovery

The two sets of key genes that were obtained using machine learning algorithms and topological parameter-based classification were grouped up- and down-regulated genes to query the cMap database, which retained potential drugs with *p*-values of less than 0.05. Drugs that were output by cMap were mapped, and finally identified with known drug targets in the up- or down-regulated cancer PPI network.

Combining datasets raises some issues, such as the problem of data heterogeneity, varying sample sizes, and the problem of data dependence. In principle, these issues can be resolved using meta-analysis. Meta-analysis [35, 36] is a set of statistical methods for summarizing the results of several investigations as a single value. The advantage of meta-analysis is that it can identify relationships across many studies.

In this drug prediction study, a *p*-value and an enrichment score (ES) are obtained for each cMap drug. The Fisher summary statistical method [36] uses the *p*-values, defined as,

1 $$ {F}_i=-2{\displaystyle \sum_{j=1}^N \log \left({p}_{ij}\right)} $$

where *F*_*i*_ tests (*χ*^2^ test with 2 *N* degrees of freedom, where *N* is the sample size) the null hypothesis for gene *i*, and indices *i* and *j* indicate the *i*th gene in the *j*th dataset respectively.

The ΕS value lies between −1 and 1, and so can be treated as a sample correlation coefficient and an index of the size of effect in the meta-analysis [36]. In practice, the ΕS value is converted to a value on Fisher’s *z* scale, and all analyses are performed using such converted values. After the analyses are completed, the *z* values are converted back to the original scale [6]. The ES value is transformed to a z value by,

2 $$ z=\frac{1}{2} \ln \frac{1+ES}{1-ES} $$

and the variance of *z* is defined as V_z_ = 1 / (*N* – 3), where *N* is the sample size. The variance of z is approximately proportional to *N*-3 (as proved by R. A. Fisher), which is independent of the value of the correlation among the population from which the sample drawn [37].

The weight that is assigned to each study in a fixed-effect model is given by,

3 $$ {W}_i=\frac{1}{V_{Y_i}} $$

where *W*_*i*_ is the within-study variance in study *i*. The weighted mean (*M*) is computed as,

4 $$ M=\frac{{\displaystyle \sum_{i=1}^k{W}_i{Y}_i}}{{\displaystyle \sum_{i=1}^k{W}_i}} $$

For unweighted calculations, *W*_*i*_ is unity. The variance of the summary effect (*V*_*M*_) is given by,

5 $$ {V}_M={\left({\displaystyle \sum_{i=1}^k{W}_i}\right)}^{-1} $$

For unweighted calculations, the Z-score for a normal distribution is defined as,

6 $$ Z=\frac{M}{S{E}_M} $$

where *SE*_*M*_ is the standard error and equals $\sqrt{V_M}$.

For weighted calculations, the Z-score is defined as,

7 $$ Z=\frac{{\displaystyle \sum_{i=1}^k{W}_i{Y}_i}}{\sqrt{{\displaystyle \sum_{i=1}^k{W_i}^2}}} $$

Equation (7) yields the one-tailed test *p*-value. The 95 % lower and upper limits on the summary effect are computed as,

8 $$ \begin{array}{l}L{L}_M=M-1.96\times S{E}_M\\ {}U{L}_M=M+1.96\times S{E}_M\end{array} $$

The formula for the random-effects model can be found in a monograph that was written by Borenstein [36]. The above analyses enable the confidence interval of the ES to be determined.

The meta-analysis involves two models - the fixed-effect model and the random-effect mod-el [36]. In the fixed-effect model, only one true effect size is assumed to exist, and all differences among studies or batches are assumed to be caused by sampling errors only. In contrast, the random-effect model allows the effect size to vary among studies, and allows an effect size to be estimated for each study. This work considers both models.

A test for the homogeneity of the distribution of data was conducted. As the size of effect commonly found to vary among studies, the meta-analysis method is used herein. *Q* statistics and *I*^*2*^ statistics are used to quantify the heterogeneity, to test it, and to incorporate it into the weighting scheme. The value of *I*^*2*^ is defined as,

9 $$ {I}^2=\frac{Q-df}{Q}\times 100\% $$

where *df* is the number of degrees of freedom, and *Q* is given by,

10 $$ Q={\displaystyle {\sum}_{i=1}^k{W}_i\Big(}{Y}_i-M\Big) $$

where *k*, *W*, *Y* and *M* are the number of studies, the study weight, the size of the effect of interest in the study and the summary effect, respectively.

A *p*-value of 0.1 for *I*^*2*^ statistics is used as the threshold for statistical significance. A *p*-value of larger than or equal to 0.1 indicates little variation among batches, and that a fixed-effect model may therefore be appropriate; otherwise, the random-effect model applies [36]. The *I*^*2*^ value represents the degree of heterogeneity: an *I*^2^ of less than 25 % implies no heterogeneity, whereas a value of larger than 75 % indicates extremely high heterogeneity.

If the studies are homogenous, then they are likely to have tested the same hypothesis. If estimates are heterogeneous, then the studies probably did not test the same hypothesis. Therefore, all of the study results may not be able to be combined in a single meta-analysis. In such a case, a separate meta-analysis, such as a meta-regression analysis, must be performed for various subsets of studies [36].

### MTT™ cell viability test

To determine the effective cytotoxicity of screening drugs, MTT assay was used for cell viability and proliferation. In general, all incubated cancer cell lines (A549 and H460) were seeded in a 96-well microplate for up to 24 h dependent on the baseline growth rate. After incubation, candidate drugs were added into the plate and incubated together for 72 h. For performing the assay, 50 μl MTT solution (2 mg/ml) per well was added and incubated at 37 °C for 2 h. The 150 μl supernatant per well was then extracted and DMSO was filled to dissolve the recipe. The absorbance was set up at 570 nm and calculated by using ELISA reader (Infinite® M1000, TECAN, Switzerland). Ratio decrease comparing to the control group as 100 % viable was seemed as the inhibitory effect.

### Clonogenic assay

We use two different high clonogenic lung cancer cell lines, A549 and H460 to perform the clonogenic assay. Cells were diluted to 500 cells per well and then seeded in 6-well plates up to 10 days according to the growth rate. Each well contained 1.5 ml RPMI medium as culture condition and screening compounds were added 24 h after the seeding. For the longer duration of incubation, medium and compounds were changed every 4 days. For performing the assay, cells were washed with PBS, and then the attached colonies were fixed with acetic acid (1: 3 diluted in methanol). The fixed colonies were stained with 0.5 % crystal violet. The colonies were then counted manually after removing the excess crystal violet and rinsing with tap water.

## Results

### Microarray data analysis

In this study, multiple microarray source data were used for analysis. The Robust Multi-array Average (RMA) was used to normalize gene expression. DEGs were predicted using an adjusted *p*-value of 0.005. Integrating DEGs data with the BioGrid [38] PPI data yielded a list of binary interactions among DEGs for both up and down groups.

The fact that that the use of various microarray platforms may raise the problem of heterogeneity is a concern, which can be tackled in the following two steps; (i) select common DEGs among all platforms for further analysis, and (ii) perform meta-analysis and test heterogeneity to determine whether the fixed-effect model or the random-effect model should be used.

### Results of machine learning

In the machine learning method, every microarray dataset is processed individually. Before conducting the machine learning algorithms, the DEGs lacking of domain data or PPI data were excluded from the candidate DEGs. The input data concerned only the remaining DEGs. After the machine learning approach was implemented, only DEGs that were identified as cancer proteins by all five topological parameter-based classifiers were considered as key genes. Table 2 presents the statistical results in this stage.

**Table 2:** The number of DEGs derived from the machine learning method for each microarray dataset

Stage	GEO ID	Number of DEGs	Excluded	Net	Number of predicted key genes	Common genes
Early	GSE7670	801	350	451	259	136
	GSE10072	2835	890	1945	1173	
	GSE19804	4614	1924	2690	1697	
	GSE27262	8476	3161	5315	3310	
Late	GSE7670	1674	608	1066	511	182
	GSE10072	1656	574	1082	691	
	GSE19804	3391	1545	1846	1181	

### Results of topological parameter-based classification

To identify key genes in the up- and down-regulated networks respectively the following process was implemented. For each group of DEGs that is classified by a topological parameter, a DEG that ranks in the top 20 % in that parameter will receive a score (S) of one. Clearly, a higher score for a DEG indicates greater importance in the network. DEGs with the highest scores in each group are selected for key genes. The key genes are the union of the two sets with the highest-scoring DEGs in the up- and down-regulated networks. In this work, this stage yielded 104 and 123 key genes for the early- and late-stage NSCLC, respectively. Focusing on the top 10 % rather than 20 % yields only 41 and 56 key genes for the early- and late-stage NSCLC. Relaxing the threshold to 30 % yields 170 and 200 key genes, respectively, which are too many; therefore, top 20 % of classified genes were chosen for key genes.

### Enriched biological pathways

Pathways are annotated using DAVID and CPDB. Top-ranking pathways in REACTOME [39] and KEGG [40] with *p*-values of less than 0.05 are reported.

In the machine learning method, the selected DEGs are microarray-specific. Common DEGs were collected from all microarray datasets as the key genes for biological pathway analysis. The key genes that were selected by topological parameter-based classification of genes in up- and down-regulated networks are merged into a single set. The two sets of key genes from the different approaches are submitted to DAVID and CPDB to extract the common enriched biological pathways.

Table 3 presents the common enriched pathways for early NSCLC that are identified by the machine learning algorithms and topological parameter classification. According to Table 3, no common pathways were identified by DAVID, while some were found using CPDB. In KEGG, endocytosis, glycolysis/gluconeo-genesis, hematopoietic cell lineage and gap junction are the common enriched pathways for early-stage cancer. According to the literature, these common pathways are closely related to cancer. Among them, glycolysis/gluconeo-genesis has been identified as an enriched pathway for early-stage cancer [6]. Oncogenes and tumor suppressors are known to regulate metabolism. The mutations of oncogenes in the up-regulation of glucose transporters increase the consumption of glucose by cancer cells, increasing the rate of glucose metabolism [41, 42]. Endocytosis is closely related to cell regulation and is predicted to play an important role in human cancers [43]. Raf/MEK/ERK is typically associated with the proliferation and drug resistance of hematopoietic cells, while the activation of the Raf/MEK/ERK cascade is suppressed in some prostate cancer cell lines that have mutations at PTEN and express high levels of activated Akt [44]. Holder et al. claimed that persistent gap junction perturbation can have chronic effects, and various tumor promoters inhibit GJ intercellular communication [45]. Cancer cells typically have down-regulated levels of gap junctions, and many pieces of evidence suggest that loss of gap junctional intercellular communication is an important step in carcinogenesis [46].

**Table 3:** The common pathways by using DAVID and CPDB for early-stage NSCLC (the *p*_*M*_ *-value* and *p*_*T*_ *-value* represent the corresponding *p*-value obtained by machine learning algorithms and topological parameter-based classification)

DAVID					
KEGG			REACTOME		
pathname	*p*_*M*_ *-value*	*p*_*T*_ *-value*	pathname	*p*_*M*_ *-value*	*p*_*T*_ *-value*
NULL			NULL		
CPDB					
KEGG			REACTOME		
pathname	*p*_*M*_ *-value*	*p*_*T*_ *-value*	pathname	*p*_*M*_ *-value*	*p*_*T*_ *-value*
Endocytosis	0.01340	0.00045	Cell-Cell communication	0.02810	0.00432
Glycolysis/Gluconeogenesis	0.02330	0.00249	Glucose metabolism	0.02420	0.02000
Hematopoietic cell lineage	0.04060	0.03380	Regulation of PLK1 Activity at G2/M Transition	0.03700	0.03070
Gap junction	0.04700	0.03930	Metabolism of nucleotides	0.03940	0.03280
			Cell junction organization	0.00765	0.03590
			Platelet activation, signaling and aggregation	0.04720	0.03630

In REACTOME, cell-cell communication, glucose metabolism, regulation of PLK1 activity at the G2/M transition, metabolism of nucleotides, organization of the cell junction and platelet activation, signaling and aggregation are enriched pathways for early NSCLC. Of them, glucose metabolism is like glycolysis/gluconeo-genesis and has been previously determined to be related to cancers. Tominaga et al. [47] demonstrated that cancer-derived extracellular vesicles (EVs), which are mediators of cell–cell communication, trigger the breakdown of the blood–brain barrier, which controls the migration of cancer cells. Arid and Zhang proposed that nucleotide metabolism causes tumor progression, and considered how this pathway can be targeted for cancer therapy by inducing the senescence of cancer cells [48]. Several cell junction components have functions that are associated with cell polarity and growth control and are specifically disrupted in cancerous cells [49]. PLK1 seems to be involved in the tumor suppressor p53-related pathways. Evidence suggests that PLK1 inhibits the transactivation and pro-apoptotic functions of p53 by physical interaction and phosphorylation [50]. Additionally, in cancer growth and dissemination, complex interactions between tumor cells and circulating platelets are critical. Evidence supports a role for physiological platelet receptors and platelet agonists in cancer metastases and angiogenesis [51].

Based on the pathway annotation database, REACTOME in DAVID, Table 4 presents the common enriched pathways for late-stage NSCLC that are identified by both methods. CPDB returns more pathways than DAVID. As noted in reference to Table 4, cell cycles are the common path that is identified using DAVID, and this finding is consistent with the results of our previous work [6]. Furthermore, many common paths were observed using CPDB, and these are dominated by the cell cycle. Notably, the endocytosis pathway appears in both Tables 3 and 4, indicating that this pathway is closely related to both early-stage and late-stage NSCLC.

**Table 4:** The common paths using DAVID and CPDB for late-stage NSCLC (the *p*_*M*_ *-value* and *p*_*T*_ *-value* represent the corresponding *p*-value obtained by machine learning algorithms and topological parameter-based classification)

DAVID					
KEGG			REACTOME		
pathname	*p*_*M*_ *-value*	*p*_*T*_ *-value*	pathname	*p*_*M*_ *-value*	*p*_*T*_ *-value*
Cell cycle	0.03800	0.00140	Cell Cycle Checkpoints	0.00760	0.00872
			Cell Cycle, Mitotic	0.00100	0.02186
CPDB					
KEGG			REACTOME		
pathname	*p*_*M*_ *-value*	*p*_*T*_ *-value*	pathname	*p*_*M*_ *-value*	*p*_*T*_ *-value*
Cell cycle	0.00632	0.00004	Regulation of mitotic cell cycle	0.01500	0.00000
Inflammatory mediator regulation of TRP channels	0.03960	0.00048	APC/C:Cdc20 mediated degradation of mitotic proteins	0.01500	0.00000
Endocytosis	0.00051	0.00153	Activation of APC/C and APC/C: Cdc20 mediated degradation of mitotic proteins	0.00663	0.00001
Thyroid hormone synthesis	0.01560	0.00662	Cell Cycle Checkpoints	0.00729	0.00001
Salivary secretion	0.03120	0.01370	Cell Cycle	0.00007	0.00001
Long-term depression	0.04420	0.02330	Regulation of APC/C activators between G1/S and early anaphase	0.00177	0.00001
cGMP-PKG signaling pathway	0.02210	0.02740	G1/S Transition	0.01030	0.00002
Vascular smooth muscle contraction	0.00497	0.03440	Cdc20:Phospho-APC/C mediated degradation of Cyclin A	0.00837	0.00004
			Cell Cycle, Mitotic	0.00485	0.00014
			APC:Cdc20 mediated degradation of cell cycle proteins prior to satisfaction of the cell cycle checkpoint	0.00182	0.00014
			Mitotic G1-G1/S phases	0.00541	0.00016
			G2/M Checkpoints	0.02590	0.00023
			M Phase	0.00298	0.00119
			DNA Replication	0.00813	0.00153
			Resolution of Sister Chromatid Cohesion	0.00103	0.00297
			Mitotic Prometaphase	0.00230	0.00399
			Apoptotic cleavage of cellular proteins	0.00067	0.00572
			S Phase	0.01500	0.00762
			Mitotic Anaphase	0.02040	0.00875
			Mitotic Metaphase and Anaphase	0.00035	0.01140
			Apoptotic execution phase	0.00036	0.01170
			Synthesis of DNA	0.03370	0.01760
			Separation of Sister Chromatids	0.00540	0.01760

DNA replication, repair and checkpoint activation pathways are highly regulated and coordinated. Defects in any of these functions cause genomic instability and may lead to cancer [52]. For example, BRCA2 participates in homologous recombination and regulating the S-phase checkpoint, and mutations of deficiencies in BRCA2 are strongly associated with tumorigenesis [53].

Table 4 agrees closely with the results of our previous work [6], which also identified cell-cycle, the mitotic anaphase, DNA replication, the sister-chromatid segregation process, the Cdc20:Phospho-APC/C-mediated degradation of Cy-clin A, the M-phase and mitotic G1-G1/S phases.

Although defective apoptosis is critical to the development and progression of cancer, apoptosis is important in the treatment of cancer as it is a popular target of many treatment strategies [54].

Wong et al. [55] noted that PKG-Iα kinase activity is necessary to maintaining high levels of cAMP response element binding (CREB) phosphorylation at ser133, and promotes the formation of colonies in NSCLC cells. The gene expression signature of the responses of vascular smooth muscle contraction to serum exposure is associated with a significantly poorer prognosis in cases of human cancer, and vascular injury response is therefore potentially linked to tumor progression [56].

According to Table 4, the mitotic process and CDC20 are involved in many enriched pathways. Mitotic progression and sister-chromatid segregation are controlled by the anaphase promoting complex/cyclosome (APC/C). APC/C forms a protein complex with its mitotic co-activator, CDC20, which controls mitotic progression. CDC20 protein level may directly influence the fate of cells during prolonged mitotic arrest and its turnover rate may critically affect the response of a cancer patient to anti-mitotic therapies [6].

In summary, combining machine learning methods with the classification of topological parameters reveals many cancer related pathways, which are well supported by the literature, providing insight into key regulators of the tumorigenesis of NSCLC.

### Potential drugs for treating NSCLC and their targeted genes

Both sets of key genes that were identified by machine learning algorithms and topological parameter-based classification were analyzed using cMap to discover potential drugs. For the set of key genes from machine learning approach and individual microarray, meta-analysis was performed using the *p*-values that were obtained from cMap for an individual microarray. For example, in early-stage NSCLC, cMap outputs 1309 drugs for key genes from the microarray GSE7670. These 1309 drugs are then filtered to find those with cMap *p*-value <0.1, and 168 drugs are identified. For cMap *p*-value <0.1, the numbers of remaining drugs for the four microarray datasets of early-stage NSCLC are 168, 139, 149 and 85 respectively. A meta-analysis is then performed to integrate the four groups of remaining drugs, and nine drugs are finally extracted. IC_50_ experiments verified the therapeutic effectiveness of four of these drugs. The alternative method begins by extracting the drugs from cMap under the constraint ES < 0, yielding 597 drugs from the 1309 drugs for microarray GSE7670. Next, the meta-analysis is performed, and 383 drugs are filtered out of the 597 drugs. Finally, 60 drugs with a meta-analysis *p*-value (*p*_*MA*_-value) of less than 0.1 are kept. Table 5 shows all of these results.

**Table 5:** The number of potential drugs filtered by meta-analysis for early- and late- stage NSCLC using the enrichment score (ES) and cMap *p*-value (less than 0.1) for meta-analysis

	Early-stage		Late-stage	
	Potential drugs	IC_50_ verified	Potential drugs	IC_50_ verified
ES < 0 & cMap *p*-value <0.1	9	4	31	5
ES < 0 & cMap *p*-value <0.5	12	4	81	8
ES < 0 & *p*_*MA*_-value <0.05	25	2	23	1
ES < 0 & *p*_*MA*_-value <0.1	60	8	49	5

In Table 6, the first row presents the early- and late- stage ES and *p*-value that is used in the meta-analysis. The upper-diagonal includes the Jaccard Index (*JI*) score of the corresponding effect size and NSCLC stage. Given two sets A and B, *JI*(A,B) is defined as |A∩B|/(|A|∪|B| - |A∩B |), where |A∩B|, |A| and |B| denote the cardinality of A ∩ B, A and B respectively. In contrast, entries in the lower diagonal are the number of common drugs for the corresponding effect size and NSCLC stage. For early-stage NSCLC, there are five common drugs (*JI* is 0.078) predicted under the two kinds of effect size, whereas, 13 common drugs (*JI* is 0.152) are identified for treating late-stage NSCLC.

**Table 6:** The number of common drugs and *JI* score for early- and late-stage using the enrichment score (ES) and cMap *p*-value (less than 0.1) for meta-analysis

	Effect size	ES < 0 & *p*_*MA*_-value <0.1		ES < 0 & cMap *p*-value < 0.1	
Effect size		Early-stage	Late-stage	Early-stage	Late-stage
ES < 0 & *p*_*MA*_-value <0.1	Early-stage		0.557	0.078	0.152
	Late-stage	39		0.074	0.194
ES < 0 & cMap *p*-value < 0.1	Early-stage	5	4		0.143
	Late-stage	12	13	5	

The drugs that are predicted by the machine learning method are the union of the drugs that are predicted under the four conditions in Table 5. In contrast, the drugs that are predicted by the topological parameter-based classification are direct outputs of the cMap with a *p*-value of less than 0.05. Machine learning algorithms (topological parameter-based classification) identified 60 (17) potential drugs for treating early-stage NSCLC, among which eight (two) were validated as effective by MTT or clonogenic assay, and are presented in Table 7.

**Table 7:** IC_50_ values of potential drugs for early-stage NSCLC

Machine learning algorithms		
cMap drug name	MTT (μM)	Clonogenic (μM)
mebendazole	<1	
vorinostat	<1	
pyrvinium	<0.1	
niclosamide	>5	
nortriptyline		<10
piperlongumine	>5	
trichostatin A		
trioxysalen	>5	
Topological parameter-based classification		
cMap drug name	MTT (μM)	Clonogenic (μM)
trichostatin A	<1	
vorinostat	<1	

Machine learning algorithms (topological parameter-based classification) identified 49 (37) potential drugs for treating late-stage NSCLC, of which were five (five) were validated as effective by MTT or clonogenic assay, and are presented in Table 8.

**Table 8:** IC_50_ values of potential drugs for late-stage NSCLC

Machine learning algorithms		
cMap drug name	MTT (μM)	Clonogenic (μM)
trichostatin A	<1	
Vorinostat	<1	
withaferin A	<1	
mebendazole	<1	
piperlongumine	>5	
Topological parameter classification		
cMap drug name	MTT (μM)	Clonogenic (μM)
acepromazine		<10
nortriptyline		<10
propafenone		<10
trichostatin A	<1	
vorinostat	<1	

Table 9 lists the common drugs that were identified by both machine learning algorithms and topological parameter-based classification. Of these, two (trichostatin A and vorinostat) were determined by IC_50_ to be effective against both early- and late-stage NSCLC, respectively. These common drugs are consistent with the findings of Ref. [5]. Seven drugs, including trichostatin A, vorinostat, MS-275, scriptaid, perhexiline, (−)-MK-801, and rifabutin, of the 18 predicted potential drugs for treating NSCLC had been reported in Ref. [5]. Interestingly, the first four drugs are HDAC inhibitors. Also, we found trichostatin A also among the 18 predicted potential drugs for treating NSCLC [6]. Notably, trichostatin A is the common drug that was identified in all of the above studies. Five common drugs, including 4,5-dianilinophthalimide, perhexiline, puromycin, trichostatin A, and vorinostat, are identified for treating both early-stage and late-stage NSCLC in this study, suggesting that they may be stage-independent drugs.

**Table 9:** The common drugs identified by both two methods

Early-stage NSCLC			
4,5-dianilinophthalimide	mepacrine (quinacrine)	meptazinol	perhexiline
puromycin	trichostatin A	vorinostat	
Late-stage NSCLC			
(-)-MK-801	4,5-dianilinophthalimide	MS-275 (Entinostat)	perhexiline
puromycin	quinostatin	rifabutin	scriptaid
trichostatin A	vorinostat	Y-27632	

Some of the above common drugs have been undergoing clinical trials for NSCLC treatment, including mepacrine (clinical trial NCT01839955), MS-275 (clinical trial NCT02437136) and Vorinostat (clinical trial NCT00667082). The results in this study are consistent with our previous work [5]; both studies identified nine drugs, of which had cytotoxic effects that were validated by IC_50_ experiments. These three drugs are trichostatin A, vorinosta and nortriptyline. The potential use in lung cancer treatment warrants further exploration. Notably, Ref. [5] treated the early stage and late stage on the same footing, it is not stage-specific.

The machine learning approach has similar hit ratio to the topological parameter-based approach (early-stage: 8/60 vs. 2/17; late-stage: 5/49 vs. 5/37), as supported by in vitro IC_50_ measurements. Combining the machine learning approach with the topological parameter-based classification yielded the best hit ratio. The current method has a higher prediction accuracy (early-stage: 2/7 vs. 1/7; late-stage: 2/11 vs. 7/65) than the method of Ref. [6], consistent with the IC_50_ measurements.

The common drugs were submitted to DrugBank [57] and NCBI to search for their corresponding targeted genes. Among these targeted genes, we kept only those which are also key genes, finally yielding a total of 8 and 12 targeted genes for early- and late-stage NSCLC respectively, as shown in Table 10, which are the potential therapeutic targets for future lung cancer clinical trials. For each targeted gene in Table 10, the number in parentheses is the number of associated cMap drugs, and could be regarded as a metric for prioritizing the genes in the list. The *ADRB2* gene ranked top of the lists for both early- and late-stage NSCLC.

**Table 10:** The targeted genes identified by the common drugs derived from both two methods (the parentheses represent the number of associated cMap drugs)

Early-stage NSCLC			
ADRB2 (4)	CASP1 (3)	KAT2A (2)	SNCA (1)
ARRB1(1)	PSIP1 (1)	PAFAH1B3 (1)	GAPDH (1)
Late-stage NSCLC			
ADRB2 (6)	ARRB1 (3)	NCOA1 (3)	PSIP1 (3)
SMARCA2 (3)	GAPDH (3)	CPT1A (1)	AURKB (1)
IRAK1 (1)	GRK5 (1)	SRPK1 (1)	AURKA (1)

**Fig. 2:**
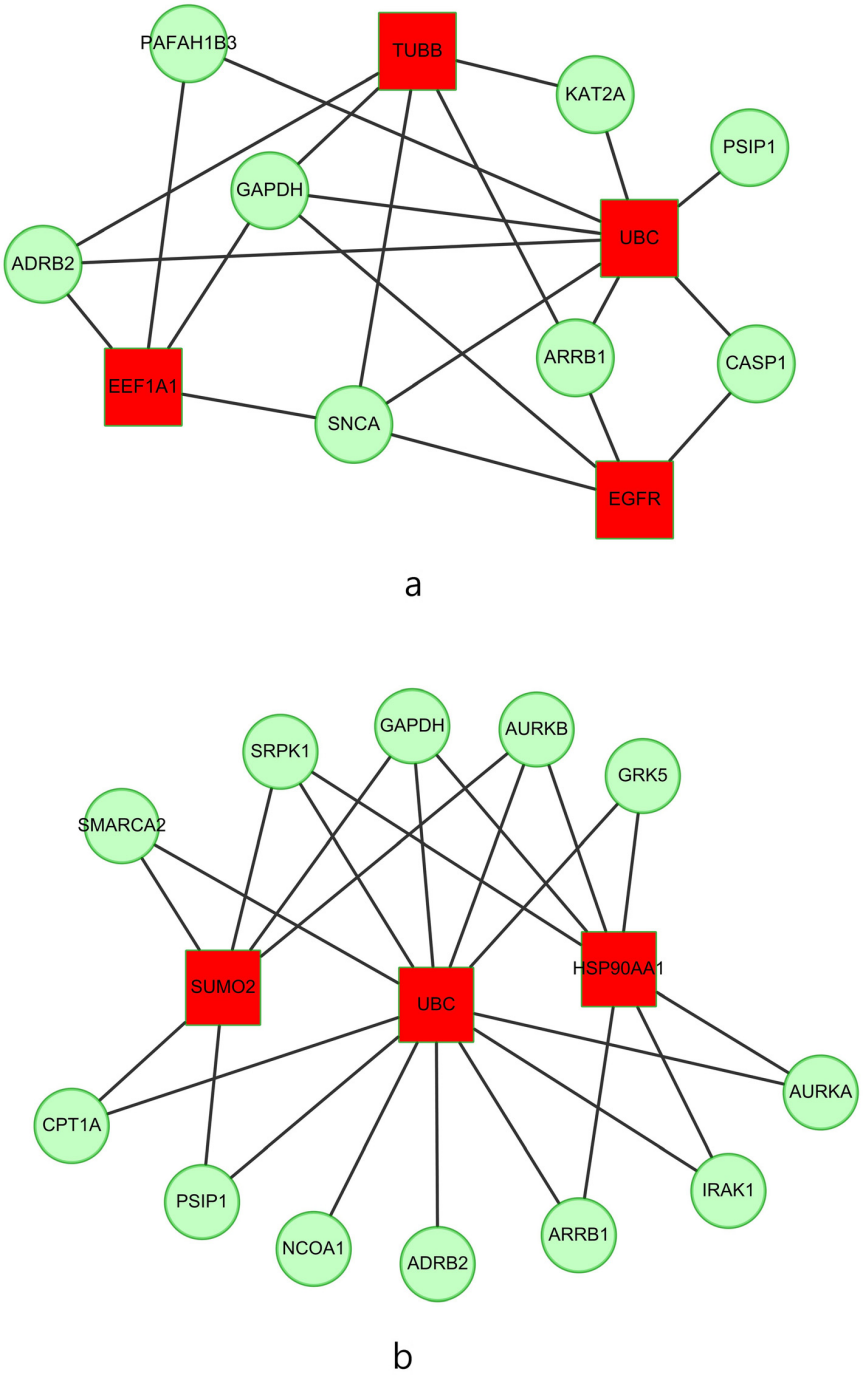
The top three genes (*squares*) which connect to the largest, the second largest and the third largest degree of targeted genes (*circles*) for **a** early-stage; **b** late-stage network

Whether a particular gene is related to most of the targeted genes in Table 10 is of interest. Therefore, networks of the targeted genes and their adjacent genes in PPI for early- and late- stage NSCLC were constructed. Figure 2a and b display the top three genes that exhibit the largest, the second largest and the third largest degree in early- and late-stage networks, respectively. In the early-stage network, the *UBC* gene directly interacts with all of the 8 targeted genes, as shown in Fig. 2a, while the other genes (such as *TUBB*) connect to no more than 5 targeted genes. Similarly, in the late-stage network, the *UBC* gene connects to all of the 12 targeted genes as shown in Fig. 2b, while the others (such as *HSP90AA1*) connect to at most 7 targeted genes. This finding is in agreement with the findings of [5]. Although the *UBC* gene is neither a key gene nor a targeted gene, it dominates all of the targeted genes; whether this fact implies that the *UBC* gene acts as a master regulator in the cancer pathway deserves further experimental investigation.

A concern arises regarding how the *p*-values that are obtained by different methods are combined. In fact, only the *p*-values and the enrichment scores (ES) that were obtained from cMap are combined in meta-analysis. Please refer to the workflow in Fig. 1. Four *p*-values were obtained by (1) identification of DEGs, (2) gene set enrichment analysis, (3) cMap drug analysis and (4) meta-analysis of cMap drugs.

The *p*-values that were obtained in the DEG analysis are used to identify significant DEGs. Also, the *p*-values that were obtained in (2) and (3) are not related to each other, and they do not have to be combined. Since different microarray datasets yielded different drug predictions, meta-analysis was conducted using the cMap *p*-values and ES to achieve results in which confidence is high.

Some missense mutations and non-synonymous SNPs (nsSNPs) may damage protein functions, disrupting the drug actions. Our future work will account for this effect. Numerous web-based tools are available to facilitate such analysis. PolyPhen2 [58] is a tool that predicts the impact of an amino acid substitution on the function and structure of a protein using sequence-based and structure-based features. SNPdryad [59] is a web-based tool that elucidates the effect of nsSNPs based on multiple sequence alignments of orthologous proteins. MutationTaster [60] is another tool that uses NGS data to elucidate the effect of missense mutations on the expression and function of proteins.

## Conclusion

In this study, two methods - machine learning algorithms and topological parameter-based classification - are compared and combined to identify potential reliable drugs for treating NSCLC, and meta-analysis is used to solve the problem of data heterogeneity. Since cancer is a multistage progressive disease, early- and late-stage cancer-related genes potentially differ substantially. Therefore, the proposed method was used to identify stage-specific DEGs, biological pathways and potential drugs. Some of the extracted biological pathways are supported by the literature, and some of the results herein concerning the identified drugs are supported by IC_50_ experiments. Seven and 11 potential drugs are discovered for treating early- and late-stage NSCLC, respectively, and warrant further investigation. Among them, perhexiline and trichostatin A are supported by the previous research. Interestingly, the *UBC* gene dominates all of the targeted genes associated with early- and late-stage NSCLC, so its role in the cancer pathway warrants further investigation.

Integrating machine learning algorithms and topological parameter-based classification herein increased drug prediction accuracy over that achieved in any previous research. This improvement is confirmed by IC_50_ experiments. The overlap of our discovered drug candidates with those that are undergoing clinical trials or are identified in the literature demonstrates the effectiveness of the proposed methods. The performance of the proposed methods can be further improved by incorporating more microarray datasets or verified gene-drug associations. In summary, many techniques were integrated to develop a novel pipeline of therapeutic drugs for NSCLC, and the efficiency of this pipeline was investigated. The approaches that were developed in this work are expected to inspire future studies, and the pipeline may be extended to the treatment of other diseases.
